# Involvement of the Reck tumor suppressor protein in maternal and embryonic vascular remodeling in mice

**DOI:** 10.1186/1471-213X-10-84

**Published:** 2010-08-06

**Authors:** Ediriweera PS Chandana, Yasuhiro Maeda, Akihiko Ueda, Hiroshi Kiyonari, Naoko Oshima, Mako Yamamoto, Shunya Kondo, Junseo Oh, Rei Takahashi, Yoko Yoshida, Satoshi Kawashima, David B Alexander, Hitoshi Kitayama, Chiaki Takahashi, Yasuhiko Tabata, Tomoko Matsuzaki, Makoto Noda

**Affiliations:** 1 Department of Molecular Oncology, Kyoto University Graduate School of Medicine, Sakyo-ku, Kyoto 606-8501, Japan; 2 Laboratory for Animal Resources and Genetic Engineering, RIKEN Center for Developmental Biology, 2-2-3 Minatojima Minami, Chuou-ku, Kobe 650-0047, Japan; 3 Laboratory of Cellular Oncology, Korea University Graduate School of Medicine, Ansan, Gyeonggi do 420-707, Korea; 4 Department of Pharmacotherapeutics, Faculty of Pharmaceutical Sciences, Doshisha Women's College of Liberal Arts, Kodo, Kyotanabe 610-0395, Japan; 5 Nagoya City University Graduate School of Pharmaceutical Sciences, 3-1 Tanabedohri, Mizuho-ku, Nagoya 467-8603, Japan; 6 Cancer Research Institute, Kanazawa University, Kakuma-cho, Kanazawa, Ishikawa 920-1192, Japan; 7 Department of Biomaterials, Institute for Frontier Medical Sciences, Kyoto University, 53 Kawahara-cho Shogoin, Sakyo-ku, Kyoto 606-8507, Japan

## Abstract

**Background:**

Developmental angiogenesis proceeds through multiple morphogenetic events including sprouting, intussusception, and pruning. Mice lacking the membrane-anchored metalloproteinase regulator Reck die *in utero *around embryonic day 10.5 with halted vascular development; however, the mechanisms by which this phenotype arises remain unclear.

**Results:**

We found that Reck is abundantly expressed in the cells associated with blood vessels undergoing angiogenesis or remodelling in the uteri of pregnant female mice. Some of the Reck-positive vessels show morphological features consistent with non-sprouting angiogenesis. Treatment with a vector expressing a small hairpin RNA against Reck severely disrupts the formation of blood vessels with a compact, round lumen. Similar defects were found in the vasculature of *Reck*-deficient or *Reck *conditional knockout embryos.

**Conclusions:**

Our findings implicate Reck in vascular remodeling, possibly through non-sprouting angiogenesis, in both maternal and embyornic tissues.

## Background

Developmental angiogenesis proceeds through multiple morphogenetic events including sprouting, intussusception (splitting of pre-existing vessels by tissue pillars), and pruning [1-3]. Several key molecules in sprouting angiogenesis have been identified: e.g., vascular endothelial growth factor (VEGF) family members and their receptors, Notch and its ligand Delta-like ligand 4 (Dll4), and semaphorins and their receptors plexin/neuropilin complexes [4-6]. Little is known, however, about the molecular bases of intussusception and pruning.

**Figure 1:**
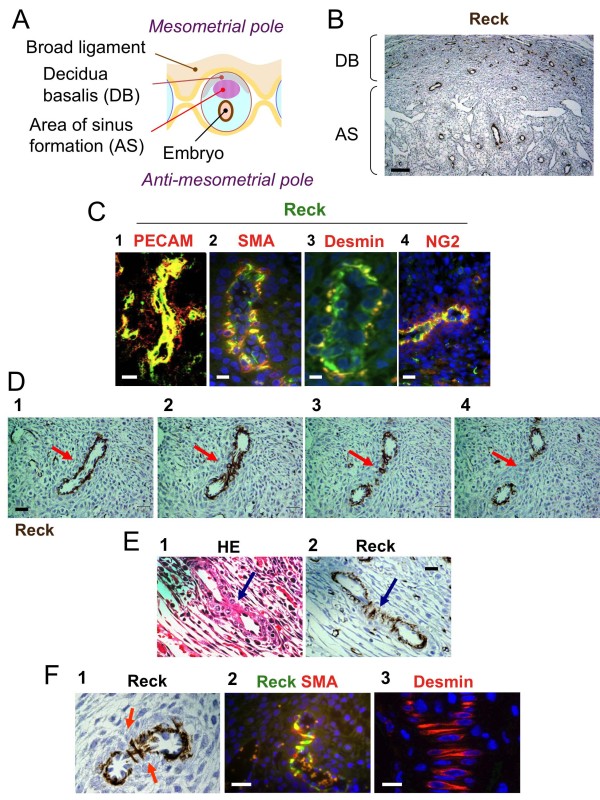
**Reck-immunoreactivity associated with blood vessels in the mouse implantation chamber**. (A) Distinct domains in the mouse implantation chamber at around 7 dpc. (B) Reck-immunoreactivity (dark brown) in the AS and DB in a longitudinal section of a 7-dpc mouse implantation chamber. (C) Loop-shaped structures in DB sections doubly stained for Reck (green) and an endothelial cell marker (red) [PECAM; panel 1] or a mural cell marker (red) [SMA, desmin, or NG2; panels 2, 3, 4, respectively] followed by nuclear counter-staining with DAPI (blue signals; panels 2-4). (D) An example of bifurcating vessels in the DB found in serial sections (4 μm-thick) stained for Reck. Red arrows indicate protruding vessel walls (panel 1) which form a contact zone (panel 2) and eventually separate the vessel into two smaller tubes (panels 3, 4). (E) An example of characteristic Reck-positive cells associated with the contact zone. Two adjacent sections were stained with hematoxylin-eosin (H&E) (panel 1) and immuno-stained for Reck (panel 2), respectively. Blue arrows indicate the contact zone. (F) The wedge-shaped cells lying across the contact zone are positive for Reck (panel 1), SMA (panel 2; fluorescent double staining with Reck), and desmin (panel 3; fluorescent staining). Scale bar: B, 100 μm; C, E, F, 20 μm; D, 30 μm.

The uteri of pregnant mice are among the most active sites of physiological angiogenesis in adult mice. Around 7 days post-coitum (7 dpc; the day when the copulation plug was confirmed is considered as 0.5 dpc in this study), the implantation chambers around the embryos are established as several swellings along the uterine horns, and in these swellings, active and precisely controlled tissue remodeling, termed decidualization, takes place [7-9]. The remodeling is particularly active in the area closer to the broad ligament (i.e., mesometrial pole) where the placenta will eventually form. In terms of histology, two distinct compartments of decidua, which we term in this paper "area of sinus formation" (AS) and "decidua basalis" (DB), respectively, become evident by 7 dpc (Figure 1A). Concomitant with the remodeling, decidual tissue acquires a complex network of newly formed vasculature that undergoes constant modification to meet the demands of the growing embryo [10]. Previous studies have implicated various growth factors (e.g., VEGF, basic fibroblast growth factor), extracellular matrix (ECM) components, and matrix metalloproteinases (MMPs) in the regulation of decidual remodeling [11]. In particular, Mmp2 and Mmp9 show dynamic expression patterns in the implantation chamber and are required for proper decidual remodeling; endogenous MMP inhibitors, such as TIMP-3, have also been implicated in the regulation of decidual remodeling [12,13]. The mechanism by which certain blood vessels are selectively preserved during this highly destructive process is yet to be elucidated.

Reversion-inducing cysteine-rich protein with Kazal motifs (*RECK*) was initially identified as a cDNA clone inducing morphological reversion (flat reversion) in NIH3T3 cells transformed by the *v-*K-*ras *oncogene [14]. *RECK *encodes a membrane-anchored metalloproteinase regulator [14-17] that is down-regulated in many cancer cells [18]. *RECK *suppresses tumor angiogenesis, invasion, and metastasis when artificially expressed in tumor cells [14,15]. Accumulating evidence indicates that RECK is down-regulated in various solid tumors and that the level of residual RECK expression in resected tumors often correlates with better prognosis, supporting the authenticity of RECK as a clinically relevant tumor suppressor [18]. *RECK *has also been implicated in the regulation of several developmental processes, including embryonic angiogenesis, myogenesis, chondrogenesis, neurogenesis, and maturation of neuro-muscular junctions [15,19-22]. In particular, *Reck*-deficient mice die around embryonic day 10.5 (E10.5) with their vascular network arrested at the stage of primary capillary plexus, suggesting angiogenesis rather than vasculogenesis is affected in these animals [15]. It is still unclear, however, why embryonic angiogenesis fails to proceed in the absence of Reck while tumor angiogenesis is suppressced by Reck. In addition, due to the midgestation lethality of the global *Reck*-deficient mice, it is yet to be explored how Reck functions during later stages of embryogenesis and in adult animals.

In this study, we found abundant expression of Reck in the cells associated with blood vessels undergoing rapid remodeling in the mouse implantation chambers. Our histological and experimental evidence implicates Reck in vascular remodeling which may involve non-sprouting mechanisms such as intussusception and pruning.

## Results

### Reck is abundantly expressed in the cells associated with remodeling blood vessels in the mouse implantation chamber

To gain insights into the functions of Reck in adult mice, we examined its tissue distribution by immunohistochemical techniques. The most abundant Reck signals were found in multiple cell types in the uteri of pregnant mice (Additional files 1, 2 and 3). In the medial longitudinal sections of the implantation chambers at 7 dpc, Reck-immunoreactivity was associated with several loop-shaped structures in the AS as well as DB and with numerous solitary cells in the DB (Figure 1A, B). Most of the loop-shaped structures consisted of an inner cell layer positive for the vascular endothelial cell marker PECAM (Figure 1C, panel 1) and an outer cell layer(s) positive for mural cell markers, i.e., α-smooth muscle actin (SMA), desmin, and NG2 (Figure 1C, panels 2-4). Reck signals seemed to be associated with both of these cell types (note the yellow signals in Figure 1C). RECK expression in these two cell types could be demonstrated by immunoblot assay using cultured human vascular endothelial cells (HUVECs) and vascular smooth muscle cells (VSMCs) (data not shown). We therefore conclude that the Reck-positive loop-shaped structures represent blood vessels. Most of the Reck-positive solitary cells, on the other hand, were associated with capillary-like structures and were positive for PECAM (Additional file 4), suggesting that they represent a sub-population of capillary-forming endothelial cells.

As we examined the serial sections of implantation chambers, two types of Reck-positive vasculature were found. In one type (Figure 1D), the vessel is banana-shaped in one section (panel 1); its opposing walls progressively protrude in subsequent sections (panel 2); and it eventually bifurcate into two tubes (panel 4). In critical sections, central regions of the protruding walls apose each other to form a contact zone like a crushed rubber tube (Figure 1E, panel 1), and a group of wedge-shaped Reck-positive cells are lying across the contact zone (Figure 1E, panel 2). These wedge-shaped cells are positive for SMA and desmin (Figure 1F). We also found some vessels bifurcating on both sides (e.g., Additional file 5A) or forming a contact zone only in the central region along their longitudinal axes (e.g., Additional file 5B).

**Figure 2:**
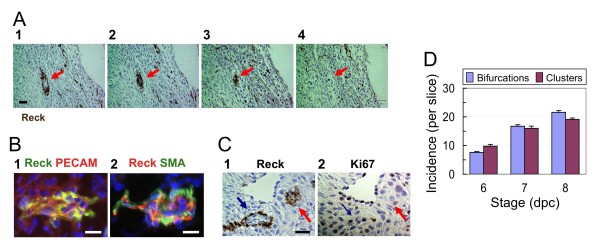
**Reck-immunoreactivity associated with clusters of vascular cells in the mouse implantation chamber**. (A) An example of a cluster of cells (red arrow) positive for Reck at a vessel terminus found in the DB. (B) Immunofluorescent double staining of a terminal cell cluster for Reck (green) and PECAM (red; panel 1) or Reck (red) and SMA (green; panel 2) followed by nuclear counter-staining with DAPI (blue). (C) Two adjacent sections stained for Reck (panel 1) and Ki67 (panel 2), respectively. Blue arrows: a bifurcating vessel. Red arrows: a terminal cell cluster. (D) Temporal changes in the number of Reck-positive bifurcating vessels (blue bar) and terminal cell clusters (red bar) per slice from the medial part of implantation chambers. Bar represents mean ± s.e.m., n = 7. The difference between stages is statistically significant (p < 0.05) for both Reck-positive bifurcating vessels and terminal cell clusters. Scale bar: A, 30 μm; B, C, 20 μm.

In the second type of Reck-positive vessel (Figure 2A), a heavily labeled tube is found in one section (panel 1); its lumen continuously narrows in subsequent sections (panels 2, 3); and it eventually disappears (Figure 2A, panel 4). Such terminal cell clusters positive for Reck (e.g., Figure 2A, panel 2, arrow) contain both endothelial and mural cells (Figure 2B).

Although some cells associated with the bifurcating vessels are positive for the proliferation marker Ki67 (Figure 2C, blue arrow), the terminal cell clusters were negative for Ki67 (Figure 2C, red arrow). These two types of Reck-positive structures progressively increased from 6 dpc to 8 dpc, a period of active vascular remodeling, in the DB (Figure 2D). These data indicate that Reck is expressed adundantly not only in embryonic vasculature but also in the adult vasculature undergoing rapid remodeling.

### *Reck *shRNA interferes with vascular remodeling in the implantation chamber

**Figure 3:**
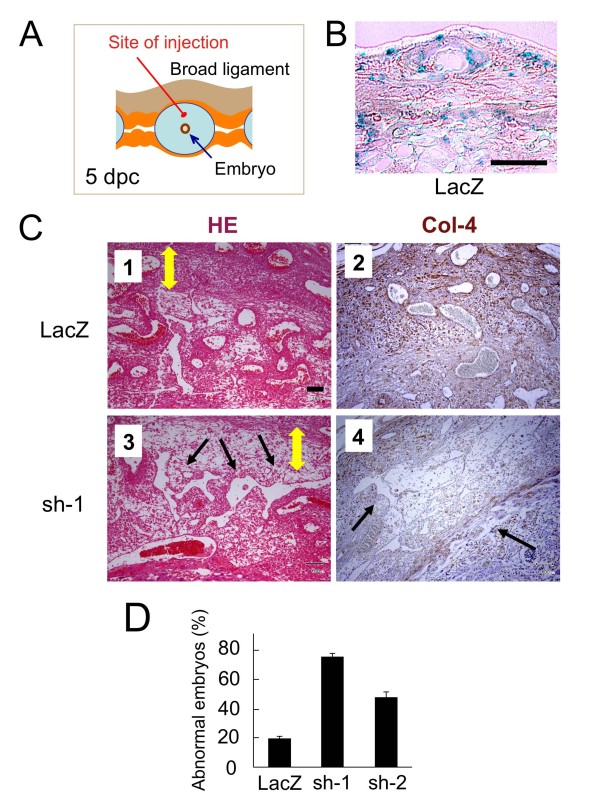
***Reck *shRNA interferes with vascular remodeling in the mouse implantation chamber**. (A) The site of bead injection (red line) in the mesometrial area at 5 dpc. (B) Control experiments to assess the efficiency of gelatin-bead-mediated gene transfer into the implantation chamber. Cationized gelatin beads impregnated with a LacZ-expression vector were injected into the mesometrial area at 5 dpc, and tissue slices were prepared at 10 dpc and stained with X-gal. A typical image is shown. (C) Mesometrial tissue at 10 dpc that had been transfected on 5 dpc with a plasmid expressing either LacZ (panels 1, 2) or shRNA against *Reck *(sh-1; panels 3, 4) was sliced and stained with H&E (panels 1, 3) or immunostained for type IV collagen (panels 2, 4). The yellow double-headed arrows indicate the DB, and the black arrows abnormal vessels. (D) The frequency of samples with abnormal decidua after transfection with vectors expressing LacZ or either of the two shRNA against Reck, sh-1 and sh-2, with different efficacy. Embryos were scored abnormal when the vessels in the transfected areas were severely disrupted as shown in panels 3 and 4 in C; the abnormality was often accompanied by reduced cellularity in the areas as well. Bar represents mean ± s.e.m., n = 7 pregnant mice. Total implantation chambers tested: LacZ, 81; sh-1, 88; sh-2, 86. Student's t-test: LacZ vs. sh-1, p = 1.7 × 10^-8^; LacZ vs. sh-2, p = 1.4 × 10^-5^; sh-1 vs. sh-2, p = 1.6 × 10^-5^. Scale bar: B, 200 μm; C, 100 μm.

Next, we asked whether Reck plays any active roles in vascular remodeling in the implantation chamber. To address this question, a method that would allow efficient and localized gene transfer into this fragile organ was required. We employed cationized gelatin beads [23] that continuously release plasmid DNA into the surrounding tissues. First, to assess the efficacy of this technique, we injected beads pre-impregnated with a control plasmid expressing LacZ into the mesometrial side of the mouse implantation chamber at 5 dpc (n = 7 pregnant mice) (Figure 3A) and harvested the tissue at 10 dpc for histological examinations. LacZ signals were found in more than 50% of the cells in the area of injection (Figure 3B). We next used a plasmid expressing a small hairpin RNA targeting *Reck *(sh-1); our previous experiments indicated that the sh-1 vector reduced the level of Reck protein down to 10% when stably transfected into cultured fibroblasts [20]. Transfer of the sh-1 plasmid resulted in some winding slits in the tissue (Figure 3C, panel 3, arrows) instead of compact round vessels as found in the control (Figure 3C, panel 1) and a marked reduction in type IV collagen-immunoreactivity (Figure 3C, panel 4) as compared to the control (Figure 3C, panels 2). We also used another plasmid expressing less active shRNA (sh-2), which knocks Reck down to 30% in cultured fibroblasts [20], and found milder effects (Figure 3D). These findings suggest that a sufficient level of Reck expression is required for proper vascular remodeling and ECM accumulation in this system.

### Vascular defects in *Reck*-deficient mice

**Figure 4:**
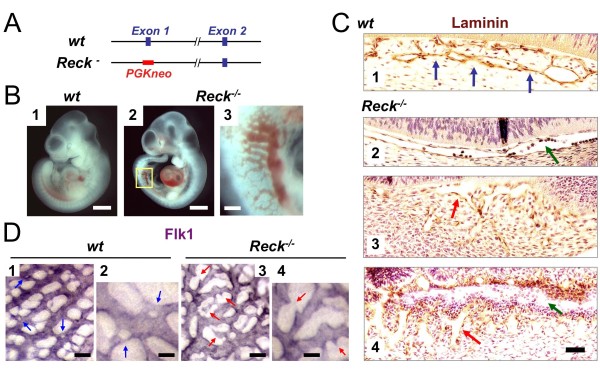
**Vascular defects in *Reck*-deficient mice**. (A) Schematic representation of the 5'-terminal region of the wild type (*wt*) and a *Reck *mutant (*Reck^-^*) allele. This mutant allele lacks exon 1 and hence no Reck protein is expressed [15]. (B) A typical E10.5 wild type embryo (panel 1) and a *Reck^-/- ^*embryo (panel 2) which shows abdominal hemorrhage and dilated vessels (panel 3, magnified view of the area indicated by yellow box in panel 2). The heart was beating in this typical mutant embryo. (C) The perineural area in the sagittal sections of wild type (panel 1) or *Reck^-/- ^*(panel 2-4) embryos stained for a basement membrane-marker, laminin. The samples in panels 2-4 represent mild, intermediate, and severe phenotypes, respectively. The blue arrows in panel 1 indicate contact zones in the wild type perineural vascular plexus, the green arrows continuous perineural vessels (panels 2, 4), and the red arrows winding vascular spaces or tissue slits (panels 3, 4). (D) Vascular networks in E10.5 wild type (panel 1; a magnified view in panel 2) or *Reck^-/- ^*(panel 3, a magnified view in panel 4) yolk sac visualized by whole-mount immunostaining for Flk-1. Blue arrows and red arrows indicate small holes and abnormal sprouts, respectively. Scale bar: B, 1 mm in panels 1, 2 and 200 μm in panel 3; C, 50 μm; D, 20 μm in panels 1, 3 and 10 μm in panels 2, 4.

These observations in maternal tissues prompted us to re-examine the morphology of vasculature in *Reck*-deficient (*Reck*^-/-^) mouse embryos. As we previously reported [15], these embryos die around E10.5 with dilated blood vessels and abdominal hemorrhage (Figure 4B, panels 2, 3), while heterozygous (*Reck*^+/-^) littermates show no obvious developmental abnormality or infertility. When vascular basement membranes in the sagittal sections of the embryos were visualized by laminin-staining (Figure 4C), a series of periodically spaced contact zones were found in the peri-neural vasculature in the wild type E10.5 littermates (Figure 4C, panel 1, blue arrows). In contrast, such contact zones were rare in the corresponding vessels of Reck-deficient littermates (Figure 4C, panels 2, green arrow). In more severe cases (Figure 4C, panels 3, 4), embryos had tissue slits with wide (green arrows) and/or winding spaces (red arrows), which is reminiscent of the tissue slits found in the implantation chamber after sh-1 transfection (see Figure 3C, panels 3, 4).

When normal yolk sac vasculature was visualized by whole-mount Flk1-immunostaining, numerous small holes were found in its arterial domain (Figure 4D, panels 1, 2, blue arrows). In the Reck-deficient yolk sacs, however, such holes were rare, and instead irregularly shaped sprouts (or branches) were abundant (Figure 4D, panels 3, 4, red arrows). In addition, arteries in the lateral region of the normal yolk sacs looked relatively straight and uniformly flattened (Figure 4D, panel 1), while those in Reck-deficient yolk sacs looked winding and variable in thickness (Figure 4D, panel 3). These findings also support the idea that *Reck *is required for proper vascular remodeling.

### Vascular defects in conditional *Reck*-deficient mice

**Figure 5:**
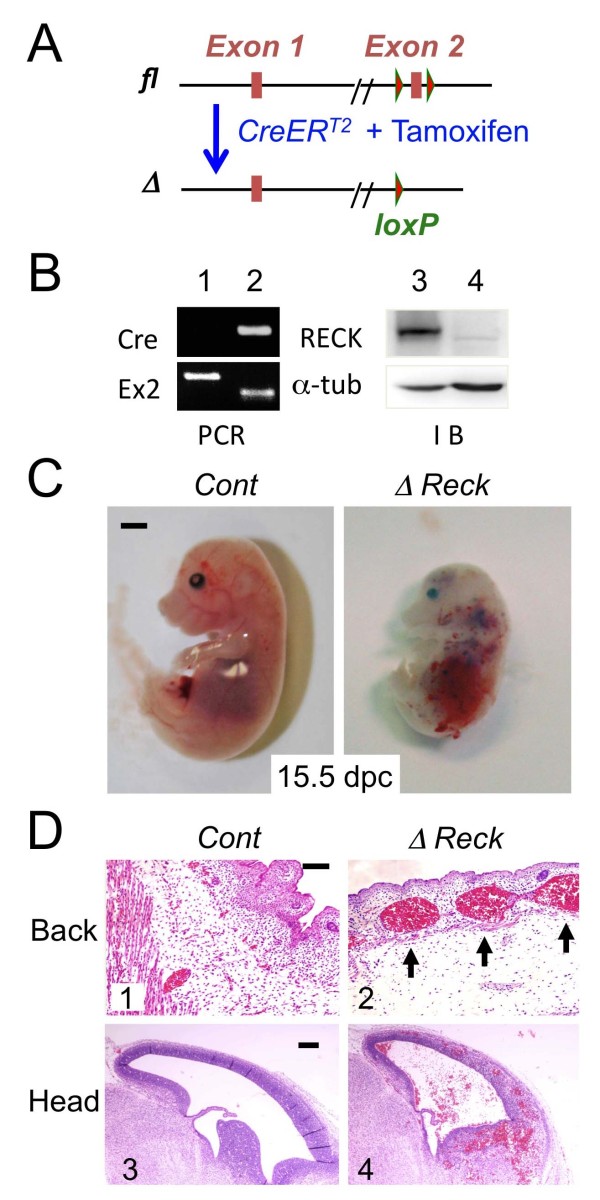
**Vascular defects in conditional *Reck*-deficient mice**. (A) 5'-terminal region of the conditional *Reck *mutant allele before (*fl*) and after (*Δ*) CreER-mediated recombination, which leads to the elimination of exon-2 and hence the early termination of translation. (B) Typical genotyping data (left panel) and Reck immunoblot assay (right panel). *Reck^fl/fl ^*females were mated with *CAG-CreER;Reck^fl/- ^*males and treated with tamoxifen from 11 dpc; embryos were harvested at E15.5. Yolk sac DNA was subjected to genotyping PCR (left panels; top, Cre primers; bottom, primers flanking *Reck *exon-2), while the whole embryo proteins were analyzed by immunoblot assay (right panels; top, Reck; bottom, α-tubulin). Note the smaller exon-2 band (lane 2, lower panel) and the absence of Reck protein band (lane 4) in the Cre-positive mouse (lane 2, upper panel). (C) Gross morphology of *Reck^fl/fl ^*(Cont) and *Reck^Δ/- ^*(*ΔReck*) embryos. The *ΔReck *embryos (i.e., *CAG-CreER;Reck^fλ/- ^*embryos treated with tamoxifen; right panel) are smaller than the control (i.e., *Reck^fλ/fl ^*embryos treated with tamoxifen; left panel) and show pale body color and haemorrhage in their heads and abdomen. The frequency of visible haemorrhage was 0% (0/16) in control animals and 100% (14/14) in *ΔReck *animals in which the hearts were beating. (D) Sagittal sections of the control (left panels) or *ΔReck *embryos (right panels) stained with H&E. Note the abnormally large blood vessels in the back region (panel 2) and the haemorrhage in the head region (panel 4) found in the *ΔReck *embryos. Scale bar: C, 1 mm; D, 100 μm.

**Figure 6:**
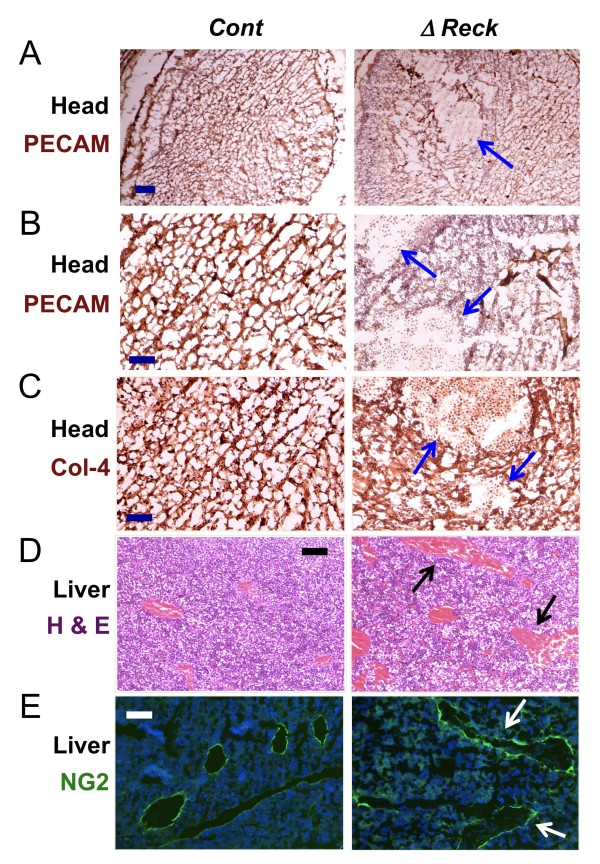
**Immunohistological anayses of conditional *Reck*-deficient mice**. Sagittal sections of the control (left panels) or *ΔReck *embryos (right panels) were stained with anti-PECAM (A, B), anti-type IV collagen (C), H&E (D), or anti-NG2 (E). NG2 in the liver (E) was visualized by immunofluorescent staining (green) followed by nuclear counter-staining with DAPI (blue). Note the abnormally large blood vessels and blood-filled cavities (arrows) found in the head (A-C) and liver (D, E) of *ΔReck *embryos. Scale bar: A 200 μm; B-E, 100 μm.

To further test this idea, we generated mice in which *Reck *expression can be conditionally silenced using a tamoxifen-inducible recombination (CAG-CreER^T2^/loxP) system [24,25] (Figure 5A). When tamoxifen-treatment (i.e., *Reck*-inactivation) was started from E11, the Cre-positive (i.e., Reck-inactivated) mice still showed embryonic lethality, indicating the continued importance of Reck during the later stages of development. These Reck-inactivated mice (Figure 5B) were still alive at E15.5, but by this time exhibited smaller-than-normal body sizes, pale body colors, and severe haemorrhage (Figure 5C). Histological examination indicated that in contrast to the compact and finely distributed vessels in the tissues of control animals (Figure 5D and Figure 6, left panels), large and irregularly shaped blood vessels, with frequently extravasated red blood cells, were found in various tissues of the Reck-inactivated mice (Figure 5D and Figure 6, right panels). Brain vasculature in these embryos were disorganized and interrupted by large spaces or cavities (Figure 6A-C, right panels, arrows), which was in sharp contrast to the fine network in the control animals (Figure Figure 6A-C, left panels). In the liver of control animals (Figure 6D, E, left panels), round and compact blood vessels tightly and evenly surrounded by NG2-positive cells (pericytes) were found. In the livers of the Reck-inactivated animals (Figure 6D, E, right panels), abnormal vessels with larger luminal spaces and loosely associated NG2-positive cells were found. Hence, the most obvious phenotype in these Reck conditional knockout mice was again vascular abnormality, albeit milder than that in the global Reck-deficient mice.

## Discussion

**Figure 7:**
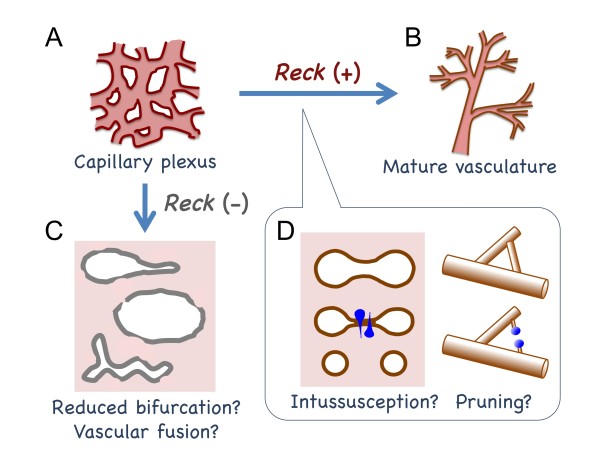
**A model on the role of Reck in vascular remodeling**. In the presence of Reck, an immature vascular plexus (A) can be remodeled into a hierarchically branched system (B). Lack of Reck results in abnormally large vessels or blood-filled cavities in the tissues (C), which may result from reduced bifurcation or excessive vascular fusion. Some of the Reck-positive cells (blue in D) may participate in vascular remodeling via non-sprouting mechanisms, such as intussusception and pruning.

Our previous study indicated that Reck expression is abundant in vascular smooth muscle cells in mouse embryos at around E10.5 and that Reck is required for embryonic angiogenesis rather than vasculogenesis [15]. In the present study, we found that abundant Reck expression is detectable in the uterus of pregnant mice (Figure 1 and Additional files 1,2,3) where adult angiogenesis is actively taking place and that abundant Reck expression can be detected in both proliferative and non-proliferative cells (Figure 2C; Additional files 1, 2). We also found that reduced, or loss of, Reck expression hinders formation of compact and organized vascular structure and instead results in tissue slits or cavities with frequent blood leakage both in the maternal (Figure 3C) and embryonic (Figure 4B, C; Figure 5C, D; Figure 6) tissues. In normal yolk sacs, small holes in the endothelial tubes, which resemble the tissue pillars in intussusception [1-3], are frequently found; in Reck-deficient yolk sacs, the small holes decrease while sprout-like structures increase (Figure 4D). These findings are consistent with the model that Reck is (or Reck-positive cells are) required for the remodeling of immature vascular plexus into a hierarchically branched system (Figure 7A, B).

In addition to the smooth muscle cells surrounding the endothelial tubes (Figure 1C), the wedge-shaped cells lying across the contact zone in bifurcating vessels were positive for Reck, SMA, and desmin (Figure 1E, F). The shape and localization of these cells are consistent with the myofibroblasts and smooth muscle cells postulated to be involved in the tissue pillar formation during intussusception [3]. The presence of vessels forming contact zones only transiently along their longitudinal axes (e.g., Additional file 5B) seems to support this model. Intusussception is known to involve less cell proliferation than sprouting and be suited for rapid formation of hierarchically branched structure in rapidly forming organs, such as embryonic lung before birth [2,3]. It is therefore tempting to speculate that intussusception occurs also in the implantation chamber where intricate placental vasculature need to be quickly generated and that Reck plays an essential role in this process (Figure 7D). To rigorously test this model, however, more fine 3D-reconstruction of serial slices or vascular cast imaging by scanning electron-microscopy is required, since occurrence of intussusception in this organ has yet to be established and it is hard to completely exclude other possibilities: e.g., we are observing bifurcation points generated by sprouting or fusion rather than splitting. Intravital imaging of Reck-positive cells may also be useful for testing this model.

It is now clear that Reck can be abundantly expressed in PECAM-positive, vascular endothelial cells (Figure 1C; Additional files 2, 3, 4). Endothelial cells are heterogeneous in morphology, function, and gene expression and retain high degree of plasticity [26]. For instance, ephrin-B2 marks the endothelium of primordial arterial vessels while its cognate receptor EphB4 marks the endothelium of primordial venous vessels [27-29]. Another example is the tip-stalk specification in sprouting vessels. Vascular spouting is guided by a subset of endothelial cells, termed tip cells, which extend filopodia and migrate actively in response to VEGF. Tip cells are followed by another subset of endothelial cells that form tubular structures in response to VEGF. Delta-like 4 (Dll4) expressed in the tip cells and its receptor Notch expressed in the stalk cells play critical role in this cell type specification [26,30]. Variety in the components of Wnt signaling pathways may also underlies further heterogeneity among endothelial cells [31]. Relationships between these different subclasses of endothelial cells and the Reck-positive endothelial cells are interesting issues that remain to be addressed.

What is the nature of the Reck-positive terminal cell clusters (Figure 2A, B)? Although these structures are located near the tips of blood vessels, they are unlikely to represent the tip cells, since they consist of multiple components of mature blood vessels (Figure 2B) rather than nascent, pioneering endothelial cells. They are more consistent with vessels undergoing regression or pruning (Figure 7D). Occurrence of active pruning in this organ, however, is yet to be established, and hence this model also needs to be rigorously tested in future studies.

What could be the machanism of action of Reck in vascular remodeling? Several hypotheses can be proposed. First, the implantation chamber is undergoing active tissue remodeling, and some areas, including the AS and DB, should be in highly destructive (or proteolytic) micro-environment. Since Reck inhibits MMPs, it may mark and help protect the blood vessels selected to be preserved. Abundant type IV collagen around the Reck-positive vessels (Additional file 6) and reduced type IV collagen after shRNA treatment (Figure 3C, panel 4) support this model. Interestingly, the area of abandant Reck expression roughly overlaps with the areas of Mmp2 and TIMP-1 expression [32], implying their interplays. Second, in light of our previous observations in other systems [19,20,33,34], Reck may be involved in more active events such as process-extension, cell-substrate adhesion, locomotion, endocytosis, and ECM accumulation/tissue consolidation. Third, Reck may hinder vascular fusion, by analogy to the activity of Reck to suppress myoblast fusion [19]. Finally, Reck may affect intracellular signaling by protecting ECM or cell surface molecules. For instance, Reck was previously found to enhance Notch-signaling by inhibiting ADAM10-mediated Notch-ligand-shedding during central nervous system development [21]. In vascular systems, Dll4 is known to suppress tip cell formation and vascular sprouting [26]. *Reck*-deficient mouse embryos seem to have increased sprouting in their yolk sac vasculature (Figure 4D, panels 3, 4), and this may support the model that *Reck*-deficiency results in attenuated Dll4/Notch signaling. It seems difficult, however, to explain the tissue slits and cavities formed after Reck knockdown/knockout solely by excessive sprouting. The phenomena look more consistent with the failure in non-sprouting vascular remodeling or excessive vascular fusion (Figure 7). In any event, Reck may shed some fresh lights on the issue of endothelial cell heterogeneity.

We previously observed that forced expression of RECK in fibrosarcoma results in reduced tumor vessel branching, increased vessel diameter, and death of tumor cells distant from expanded blood vessels [15]. Although forced expression of Dll4 in tumor cells results in decreased tumor vessel branching and increased vessel diameter, it promotes tumor growth rather than cell death [35,36]. Again, it seems difficult to fully explain the effects of RECK on tumor angiogenesis by enhanced Notch signaling. It is more likely that Reck affects multiple signaling pathways (including Notch pathway) in multiple cell types and biological outcome of altered Reck expression reflects their net effects.

Our recent studies suggest that Reck expression is regulated by several external stimuli, including serum/growth factors, cell density, and oxygen concentration [37-39]. The present study suggests that reduced Reck expression has devastating effects of both maternal and embryonic tissues, which may lead to infertility, abortion, and developmental defects in the offspring. Further studies on the roles and the mechanisms of regulation of Reck in uterine reproductive cycles and embryonic development may provide information useful for controlling these disorders.

## Methods

### Animals and tissues

The animal experiments were approved by Animal Research Committee, Graduate School of Medicine, Kyoto University and conducted following the guidelines of Kyoto University. For immunohistochemical staining, the mouse implantation chambers were fixed in 4% para-formaldehyde (PFA) for 2 h (Reck, CD31, type IV collagen, laminin, desmin, NG2, and SMA) or overnight (Ki67). Perfusion was not used in most of the experiments, for it did not make appreciable difference in vessel morphology in some earlier experiments. After fixation, paraffin embedded tissue blocks were prepared [40] and sliced (4 μm-thick). For lacZ-staining, tissues were fixed in 0.5% glutaraldehyde in phosphate buffered saline without divalent cations (PBS) at 4°C for 1 h, incubated in 30% sucrose, 2 mM MgCl_2 _for 30 min at 4°C, rinsed twice in PBS, embedded in OCT compound, and stored at -80°C until the sections (10 μm thick) were prepared using a cryostat (Leica). For immunofluorescence staining, tissues were fixed in 1% PFA, subjected to 3 steps of buffer replacements [30 min in 0.1 M glycine in PBS, 30 min in Holt's agarose-sucrose (30%) solution [22], 30 min in 1:1 mixture of OCT and Holt's agarose-sucrose solution], embedded in OCT compound, quickly frozen in dry ice, and sliced using a cryostat (7 μm-thick). Unless otherwise stated, sections were cut longitudinally near the central plane of implantation chambers. For serial section analyses, at least 20 sections were prepared from a single sample.

### DAB staining

To visualize Reck in tissues, paraffin sections were stained using the monoclonal antibody 5B11D12 [14] with the aid of the Envision+ system (Dako #K4001). Detailed procedures and the specificity of this antibody have been described elsewhere [15,22]. Other antibodies used were as follows: type IV collagen (Progen #10760), laminin (Progen, #10765), SMA (Dako, M0851), PECAM (BD Pharmingen #550274), Ki67 (Nova Castra #NCL-Ki67p), and NG2/CSPG4 (Chemicon # AB5320). The staining of deparaffinized sections was carried out with the aid of the Envision+ system for SMA and following the standard ABC protocol for PECAM, NG2, and Ki67. For PECAM staining, sections were pre-treated with Proteinase K solution (20 μg/ml in TE Buffer, pH 8.0) for 10 min at 37°C and allowed to cool for another 10 min at room temperature (RT). For Ki67 staining, sections were heated at 95°C in 0.01 M sodium citrate buffer for 20 min and allowed to cool for 20 min at RT. All sections were incubated in 3% hydrogen peroxide, 0.01% sodium azide in PBS for 30 min to block endogenous peroxidases, washed 3 times with PBS, blocked with appropriate serum for 30 min, and then incubated with primary antibodies in dilution buffer A [1% BSA (Sigma), 0.05% sodium azide in PBS] for 1 h at RT. Isotype-matched non-specific IgG was used as a negative control. Immune-complexes were then detected using the ABC system (Vector) with 3, 3-diaminobenzidine-HCl (Dako, K3467) as a chromogen.

### Immunofluorescent staining

Sections were allowed to air-dry for 20 min at RT, washed twice with PBS, treated with 20% BlockAce (Dainippon Pharmaceutical, Japan) and with appropriate serum (5%) in PBS overnight, and washed three times in PBS, followed by incubation for 1 h at RT with primary antibodies in dilution buffer B [1% BSA, 0.01% sodium azide in PBS]. The sections were then washed three times in PBS, incubated for 1 h at RT with secondary antibodies, washed three time in PBS, mounted with media containing DAPI (Vector #H-1200), and observed using a fluorescence microscope (Axioplan, Zeiss) or a confocal laser-scanning microscope (Fluoview 300, Olympus). Primary antibodies used were the same as those used in DAB staining. Isotype-matched non-specific IgG or pre-immune serum was used as a control. Secondary antibodies used are as follows: goat anti-mouse Alexa Fluor 488 (Invitrogen#A11001) for Reck and SMA, goat anti-Rat TRITC (Jackson ImmunoResearch #112 025 167) for PECAM, and goat anti-Rabbit Alexa Fluor 555 (Invitrogen#A21428) for laminin and NG2. For Reck/Desmin double staining, rhodamine-conjugate anti-mouse IgG (Jackson ImmunoResearch, #115-295-205) and FITC-conjugated anti-mouse IgG2a (Jackson ImmunoResearch, #115-095-205) were used to detect Desmin and Reck, respectively. For Reck/SMA double staining, a Zenon mouse IgG labeling kit (Invitrogen#Z25160) was employed.

### LacZ staining

Sections were brought to room temperature, washed twice with PBS, incubated overnight in X-gal solution [0.1 M phosphate buffer (pH 7.3), 2 mM MgCl_2_, 0.01% sodium deoxycholate, 0.02% NP-40, 5 mM potassium ferricyanide, 5 mM potassium ferrocyanide, 1 mg/ml X-Gal, 20 mM Tris-HCl (pH 7.3)], and then counterstained with eosin.

### Plasmid delivery into implantation chambers

Cationized gelatin beads (4 mg) prepared as described previously [23,41] were incubated in 50 μl PBS containing 40 μg plasmid DNA for 24 hr. Pregnant mice (5 dpc) were anaesthetized by intra-peritoneal injection of nembutal (50 mg/kg) and subjected to laporatomy to expose each uterine horn. The bead suspension (10 μl; ~5 μg DNA) was injected into the mesometrial area of each implantation chamber using a microsyringer. The abdominal cavity was stitch-closed with sutures (Akiyama, Japan). Five days after the operation, the animals were sacrificed for histological examinations. For each plasmid DNA, seven pregnant mice (total of more than 80 implantation sites) were used.

### Mutant mice

Generation of the global *Reck-*deficient mice and the histological analyses of the E10.5 embryos and yolk sacs have been described elsewhere [15]. Mice carrying a modified *Reck *allele, named *Reck^fl ^(R2) *(Acc. No. CDB0488K: http://www.cdb.riken.jp/arg/mutant%20mice%20list.html), in which the 2nd exon was flanked by two *loxP *sites, were generated using the bacterial artificial chromosome (BAC) clone containing the C57BL/6 genomic *Reck *sequence (RP23-9F18; BACPAC Resources, Oakland, USA) as a template and following our established protocols for constructing targeting vectors (see http://www.cdb.riken.go.jp/arg/download_file/vector_09). In brief, the 3.4 kb 5'-arm, 7.9 kb 3'-arm, and 0.4 kb exon 2-containing fragment of Reck were inserted respectively into the 5'-, 3'-, and the central cloning sites of the DT-A/Conditional FW vector http://www.cdb.riken.go.jp/arg/cassette.html (Additional file 7). Mutant ES clones were isolated after electroporation of this vector into TT2 ES cells [42], and a transgenic mouse line was established using one such clone (No. 25). The PGK-neo fragment was then eliminated by crossing this line with a mouse carrying the *Flp *transgene. The CreER transgenic mouse [STOCK Tg(CAG-cre/Esr1)5Amc/J] was obtained from The Jackson Laboratory (Bar Habor, USA). *Reck^fl/fl ^*females were mated with *CAG-CreER*;*Reck^fl/- ^*males, and tamoxifen (60 mg/kg) was injected intra-peritoneally into pregnant mice five times (24 hr interval between each injection) from 11 dpc. Mice carrying the *CAG-CreER *transgene were not found among the pups born after this treatment. The status of the *Reck *allele was assessed by PCR [primers: AGTACATGACTTAGGAACAG, AACTGCAATATCTGGGATAC; conditions: 94°C, 30 s/61°C, 30 s/72°C, 60, 35 cycles; products: 853 bp (*wt*), 1252 bp (*fl*), 721 bp (*Δ*).

### Immunoblot assay

Proteins were solubilized and subjected to immunoblot assay using the anti-RECK antibody 5B11D12 as described previously [16,22]. The blot was re-probed using antibodies against the loading control, α-tubulin (Calbiochem).

### Statistical analyses

To count the numbers of Reck-positive capillaries, bifurcation points, and terminal cell clusters in the DB, a total of seven implantation chambers were randomly selected from 3 pregnant mice at each stage (i.e., 6, 7, and 8 dpc). Five longitudinal sections (4 μm-thick) near the central plane of each implantation chamber were prepared from each chamber. The number of structures found in five sections from each implantation chamber was scored, and the mean ± s.e.m. are presented. In the cases of gelatin beads treatment, all the implantation chambers from 7 mice per group were analyzed. The statistical significance of difference was assessed by Student's t-test.

## Authors' contributions

EPSC carried out all the experimental and histological studies using the mouse implantation chambers and drafted the manuscript. YY, SKa, HKit, and CT helped in these experiments. YM, AU, and RT analyzed the conditional knockout mouse embryos. HKiy, NO, MY, and TM generated the Reck-floxed mice. SKo, JO, and RT analyzed the yolk sac vasculature. YT helped in the experiments using cationized geltin beads. MN contributed in the design and coordination of this study and drafted the manuscript with the help of DBA. All authors read and approved the final manuscript.

## Supplementary Material

Additional file 1 **Reck-immunoreactivity in the inter-implantation areas of the uteri in pregnant mice**. (A) Schematic representation of a medial longitudinal section of mouse implantation chambers at 7 dpc. Relative position of the area focused in this figure is highlighted in blue. (B) Reck-positive uterine epithelium at 7 dpc. A magnified view of the area as indicated by the blue arrow in panel 1 is shown in panel 2. A pair of adjacent slices were stained for Reck (panel 3) and Ki67 (panel 4). The Reck-positive epithelium at this stage is largely non-proliferative (panels 3, 4, red arrow). (C) Reck-positive cells at 1 dpc. A magnified view of the area indicated by the blue box in panel 1 is shown in panel 2. A pair of adjacent slices were stained for Reck (panel 3) and Ki67 (panel 4). Reck-signals are largely associated with interstitial capillaries but not with the proliferative uterine epithelium at this stage (panels 3, 4, red arrow). Scale Bar: B1, B5, 100 μm; B2, 10 μm; B3 & 4, 30 μm; B6, 20 μm; B7 & 8, 50 μm. Click here for file

Additional file 2 **Reck-signals associated with strings of cells in the AS**. (A) Schematic representation of a medial longitudinal section of mouse implantation chambers at 7 dpc. Relative position of the area focused here is highlighted in blue. (B) Typical medial (panel 1; e, embryo) and lateral (panel 2) sections of a 7-dpc implantation chamber stained for Reck. Reck-signals are abundant in medial sections but not in lateral sections. In medial sections, the most prominent Reck-signals are found in the region near DB (red bracket; see Figure 1). In addition, moderately strong Reck-signals are found in two regions located symmetrically in the AS (green arrows). In these regions, the Reck-positive cells form several strings. (C) Pairs of adjacent slices stained for Reck and SMA (panels 1, 2) or Reck and PECAM (panels 3, 4). Reck-signals tend to colocalize with SMA-signals (panels 1, 2) rather than PECAM-signals (panels 3, 4). (D) Fluorescent triple staining for Reck (green), PECAM (red), and nuclei (blue). The PECAM-positive parts of the strings (see panel C4, yellow bracket) are also mildly Reck-positive. (E) Adjacent slices stained for Reck and Ki67. The Reck-positive strings of cells (panel 1) are largely non-proliferative (panel 2). Scale Bar: B, 300 μm; C1 & 2, 50 μm; C3 & 4, 100 μm; D, 20 μm; E, 10 μm. Click here for file

Additional file 3 **Reck-immunoreactivity in the AS at later stages and in the primary desidual zone (PDZ)**. (A) Schematic representation of mouse implantation chambers at 7 dpc. Relative positions of the areas focused in this figure are highlighted in blue. (B) Reck-immunoreactivity in the AS at 8 dpc. Magnified view of an area as indicated by yellow ellipse in panel 1 is shown in panel 2. The expanding sinuses are lined by Reck-positive cells. Red bracket indicates the area where larger sinuses are formed and green arrow decidua basalis (DB) where numerous Reck-positive vessels are found (see Figure 1). (C) Fluorescent triple staining of a 8.5-dpc slice for Reck (green), PECAM (red), and nuclei (blue). At this stage, Reck-positive cells show clear endothelial phenotype. (D) The AS at 9 and 10 dpc stained for Reck. Reck signals become weaker at 9 dpc (panel 1) and barely detectable at 10 dpc (panel 2). Red arrow indicates the placental bed. (E) Reck signals associated with decidual cells in the PDZ at late 7 dpc. Magnified view of the area indicated in the yellow circle in panel 1 is shown in panel 2. Blue arrow indicates the trophoblast cell layer. Red arrows indicate clusters of the Reck-positive decidual cells. Scale Bar: B1, 300 μm; B2, 50 μm; C, 25 μm; D1, 30 μm; D2, 300 μm; E1, 400 μm; E2, 50 μm. Click here for file

Additional file 4 **Reck-positive solitary cells in the DB**. (A) A magnified view of Reck-positive solitary cells in the DB. (B) DB vessels doubly immuno-stained for Reck and PECAM and observed with a confocal microscope. (C) Proportion of PECAM-positive or SMA-positive cells among the Reck-positive solitary cells in the DB (n = 7). Scale bar: A 20 μm; B 50 μm. Click here for file

Additional file 5 **Examples of Reck-positive vessels in the DB**. Serial sections of 7-dpc implantation chambers were stained with anti-Reck antibody. (A) The vessel indicated by red arrows shows a bifurcation (panels 1, 2) and contact zone (panels 3, 4) on the one side, while the vessel indicated by blue arrows shows bifurcations (panels 1, 2, 6, 7) and contact zones (panels 3, 5) on both sides along the longitudinal axis. A possible topology of the latter vessel is shown in panel 8 (not in scale). (B) The vasculature marked with blue arrows shows a contact zone only in the central region (panels 2, 3) and not on both sides (panels 1, 4-6) along the longitudinal axis. A possible topology of the vessel is shown in panel 7 (not in scale). Scale bar: 50 μm. Click here for file

Additional file 6 **Reck- and type IV collagen-immunoreactivity around blood vessels in the DB**. DB vessels in two adjacent sections were stained for Reck (A, B) and type IV collagen (C, D). Magnified views of the area indicated by yellow boxes in (A) and (C) are shown in (B) and (D), respectively. Type IV collagen is abundant around the Reck-positive cells. Scale bar: A, C 100 μm; B, D 20 μm. Click here for file

Additional file 7 **Reck targeting strategy**. See Methods for detail. PCR with primers AGTACATGACTTAGGAACAG (→) and AACTGCAATATCTGGGATAC (←) generates 853, 1252, and 721-bp products representing *wt, fl*, and *Δ *allele, respectively. Click here for file
