# Supplementary figures and images for: Involvement of the Reck tumor suppressor protein in maternal and embryonic vascular remodeling in mice
Source: BMC Dev Biol. 2010 Aug 6;10:84. doi: 10.1186/1471-213X-10-84 (PMC2923112; doi:10.1186/1471-213X-10-84)

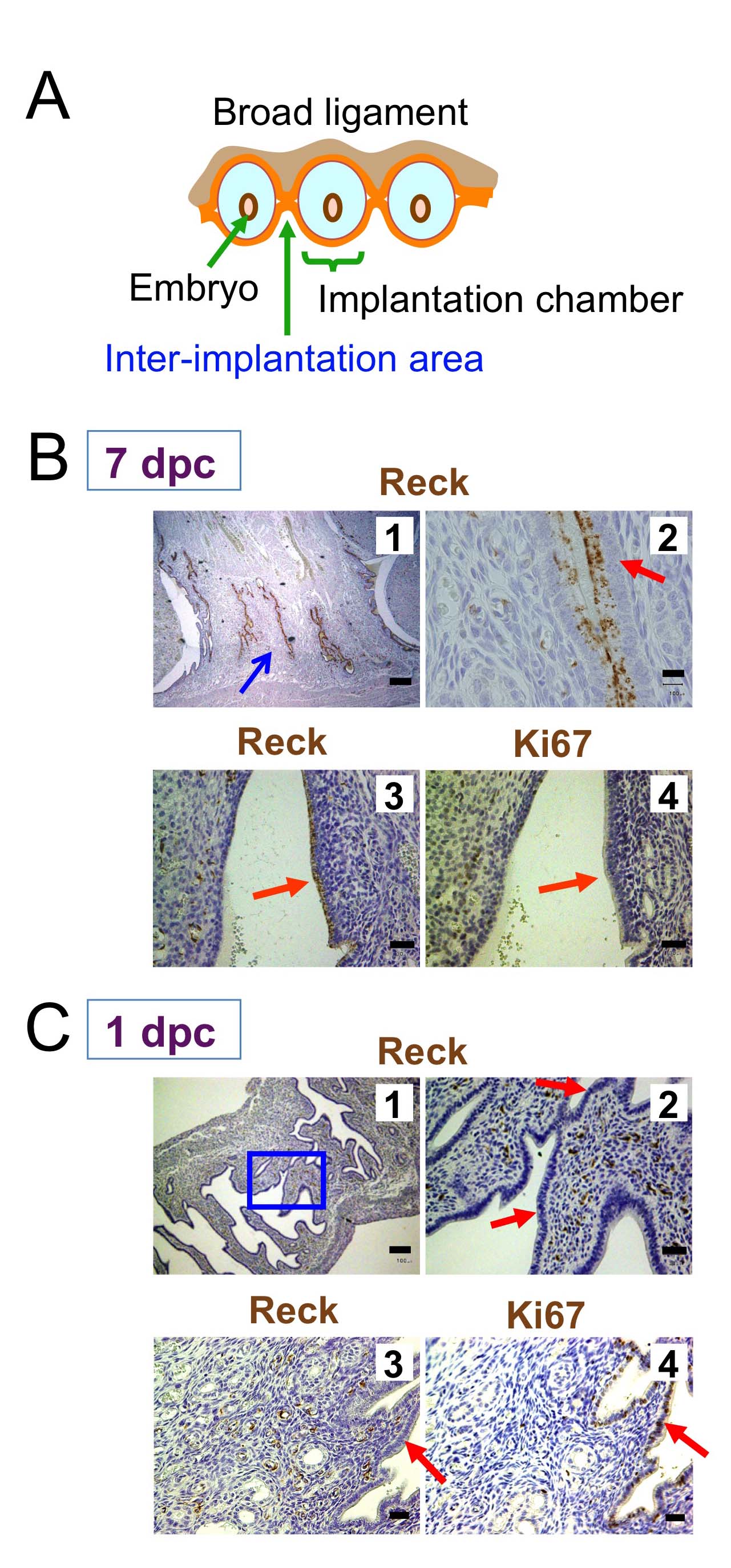

Supplement: Additional file 1 — Reck-immunoreactivity in the inter-implantation areas of the uteri in pregnant mice. (A) Schematic representation of a medial longitudinal section of mouse implantation chambers at 7 dpc. Relative position of the area focused in this figure is highlighted in blue. (B) Reck-positive uterine epithelium at 7 dpc. A magnified view of the area as indicated by the blue arrow in panel 1 is shown in panel 2. A pair of adjacent slices were stained for Reck (panel 3) and Ki67 (panel 4). The Reck-positive epithelium at this stage is largely non-proliferative (panels 3, 4, red arrow). (C) Reck-positive cells at 1 dpc. A magnified view of the area indicated by the blue box in panel 1 is shown in panel 2. A pair of adjacent slices were stained for Reck (panel 3) and Ki67 (panel 4). Reck-signals are largely associated with interstitial capillaries but not with the proliferative uterine epithelium at this stage (panels 3, 4, red arrow). Scale Bar: B1, B5, 100 μm; B2, 10 μm; B3 & 4, 30 μm; B6, 20 μm; B7 & 8, 50 μm. [file 1471-213X-10-84-S1.JPEG]

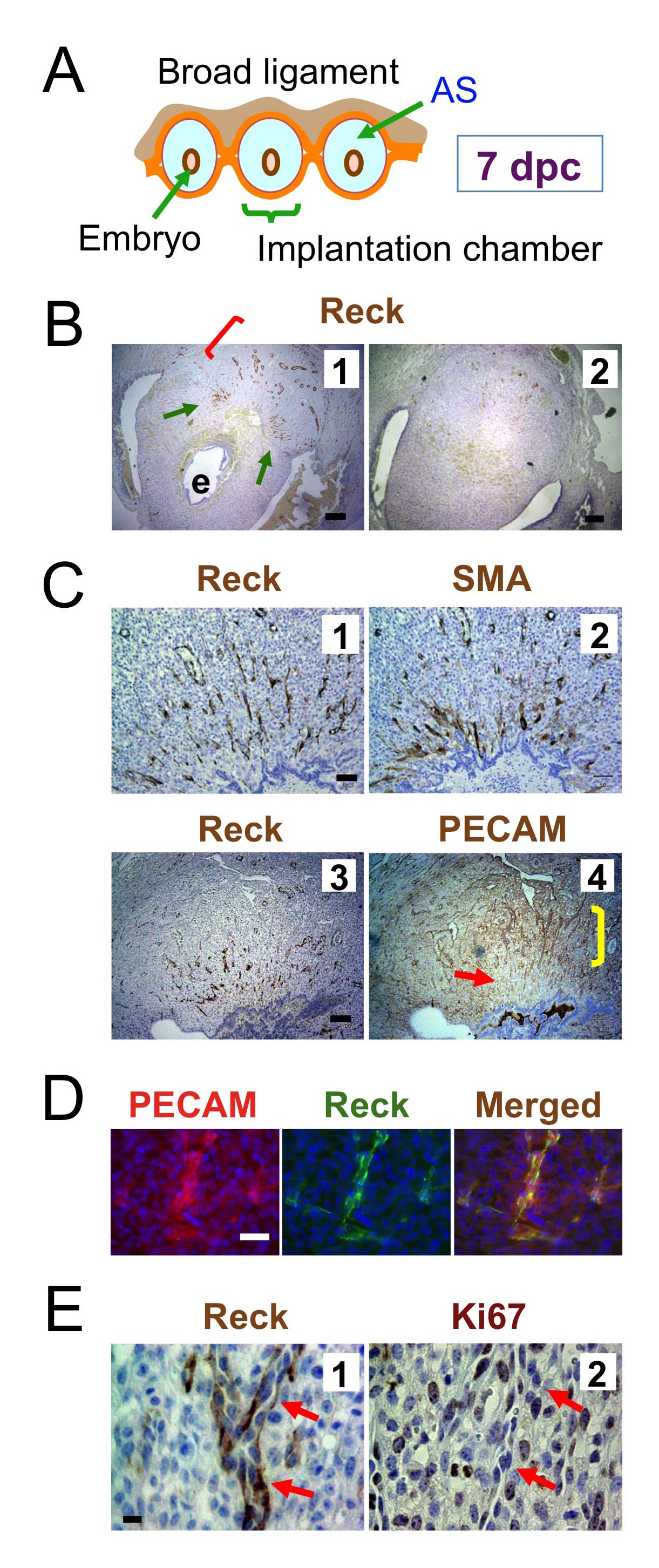

Supplement: Additional file 2 — Reck-signals associated with strings of cells in the AS. (A) Schematic representation of a medial longitudinal section of mouse implantation chambers at 7 dpc. Relative position of the area focused here is highlighted in blue. (B) Typical medial (panel 1; e, embryo) and lateral (panel 2) sections of a 7-dpc implantation chamber stained for Reck. Reck-signals are abundant in medial sections but not in lateral sections. In medial sections, the most prominent Reck-signals are found in the region near DB (red bracket; see Figure 1). In addition, moderately strong Reck-signals are found in two regions located symmetrically in the AS (green arrows). In these regions, the Reck-positive cells form several strings. (C) Pairs of adjacent slices stained for Reck and SMA (panels 1, 2) or Reck and PECAM (panels 3, 4). Reck-signals tend to colocalize with SMA-signals (panels 1, 2) rather than PECAM-signals (panels 3, 4). (D) Fluorescent triple staining for Reck (green), PECAM (red), and nuclei (blue). The PECAM-positive parts of the strings (see panel C4, yellow bracket) are also mildly Reck-positive. (E) Adjacent slices stained for Reck and Ki67. The Reck-positive strings of cells (panel 1) are largely non-proliferative (panel 2). Scale Bar: B, 300 μm; C1 & 2, 50 μm; C3 & 4, 100 μm; D, 20 μm; E, 10 μm. [file 1471-213X-10-84-S2.JPEG]

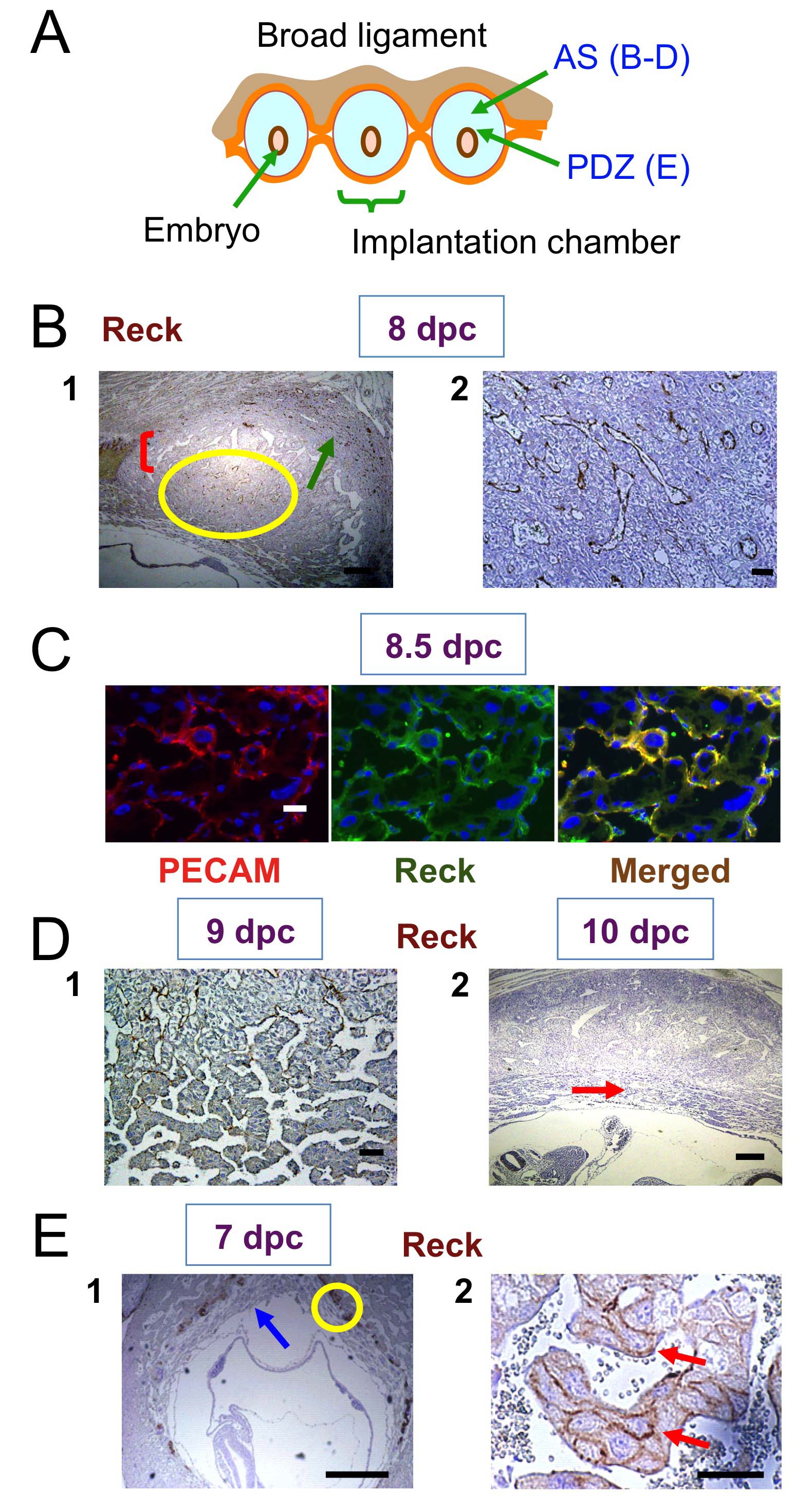

Supplement: Additional file 3 — Reck-immunoreactivity in the AS at later stages and in the primary desidual zone (PDZ). (A) Schematic representation of mouse implantation chambers at 7 dpc. Relative positions of the areas focused in this figure are highlighted in blue. (B) Reck-immunoreactivity in the AS at 8 dpc. Magnified view of an area as indicated by yellow ellipse in panel 1 is shown in panel 2. The expanding sinuses are lined by Reck-positive cells. Red bracket indicates the area where larger sinuses are formed and green arrow decidua basalis (DB) where numerous Reck-positive vessels are found (see Figure 1). (C) Fluorescent triple staining of a 8.5-dpc slice for Reck (green), PECAM (red), and nuclei (blue). At this stage, Reck-positive cells show clear endothelial phenotype. (D) The AS at 9 and 10 dpc stained for Reck. Reck signals become weaker at 9 dpc (panel 1) and barely detectable at 10 dpc (panel 2). Red arrow indicates the placental bed. (E) Reck signals associated with decidual cells in the PDZ at late 7 dpc. Magnified view of the area indicated in the yellow circle in panel 1 is shown in panel 2. Blue arrow indicates the trophoblast cell layer. Red arrows indicate clusters of the Reck-positive decidual cells. Scale Bar: B1, 300 μm; B2, 50 μm; C, 25 μm; D1, 30 μm; D2, 300 μm; E1, 400 μm; E2, 50 μm. [file 1471-213X-10-84-S3.JPEG]

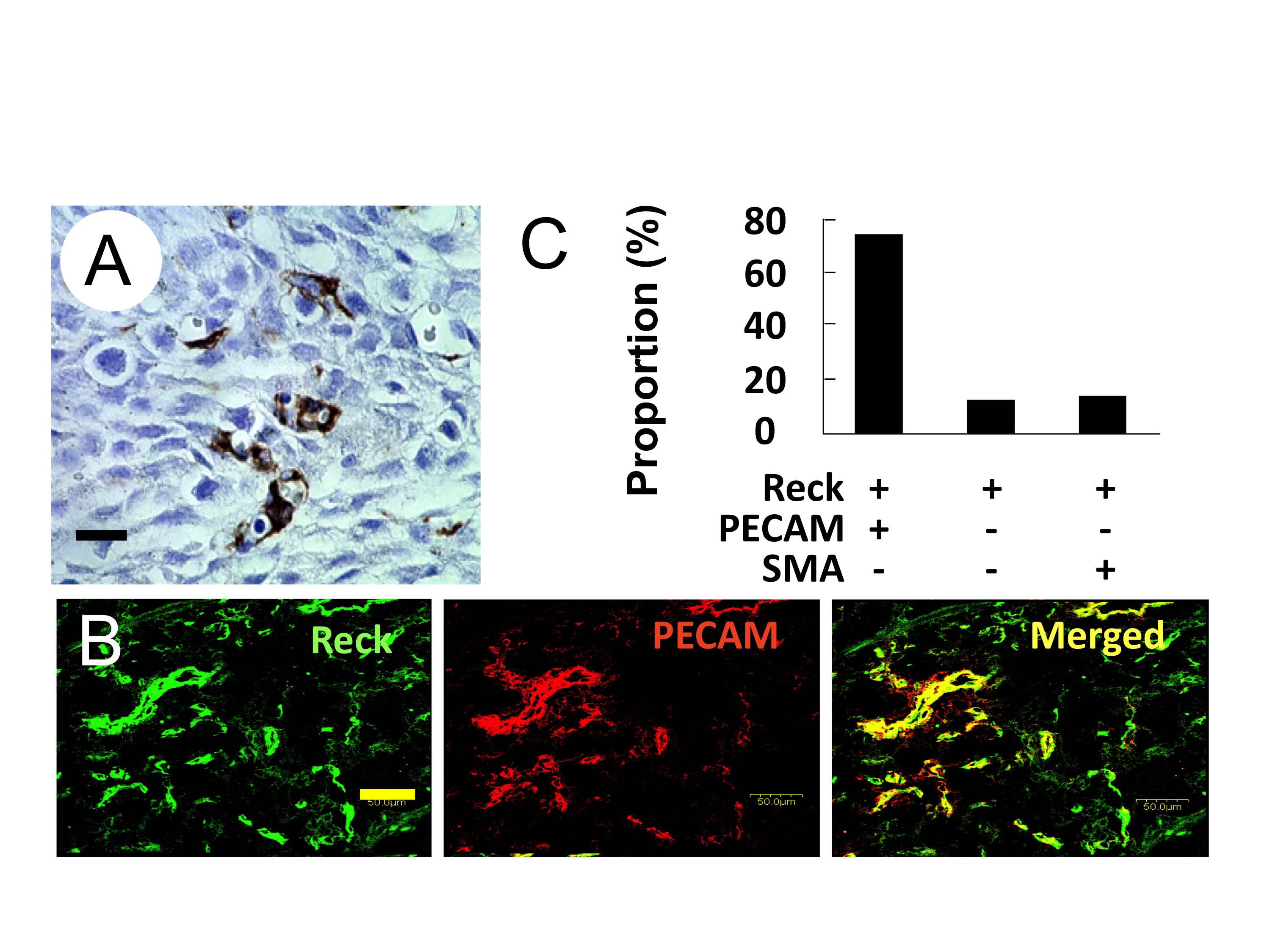

Supplement: Additional file 4 — Reck-positive solitary cells in the DB. (A) A magnified view of Reck-positive solitary cells in the DB. (B) DB vessels doubly immuno-stained for Reck and PECAM and observed with a confocal microscope. (C) Proportion of PECAM-positive or SMA-positive cells among the Reck-positive solitary cells in the DB (n = 7). Scale bar: A 20 μm; B 50 μm. [file 1471-213X-10-84-S4.JPEG]

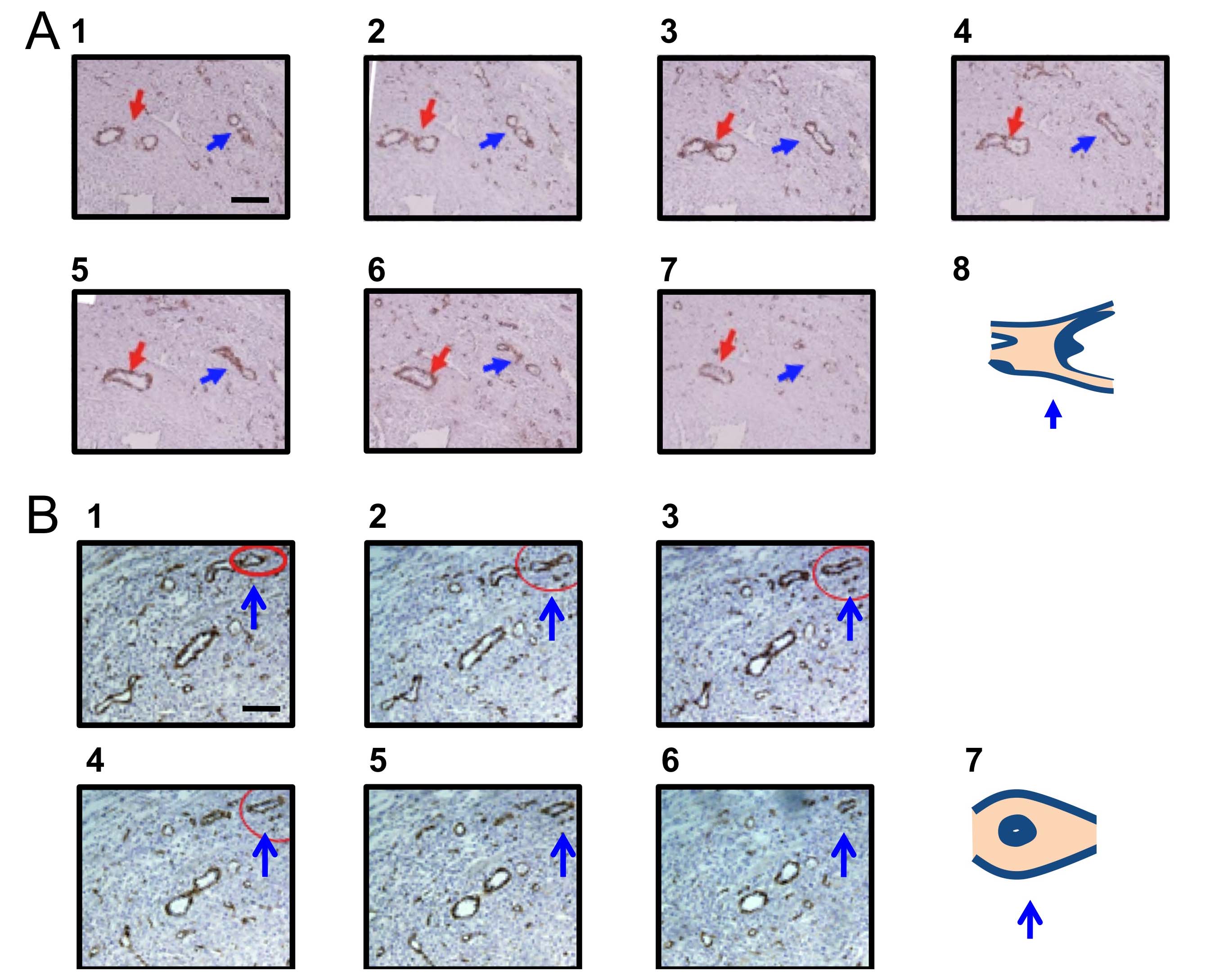

Supplement: Additional file 5 — Examples of Reck-positive vessels in the DB. Serial sections of 7-dpc implantation chambers were stained with anti-Reck antibody. (A) The vessel indicated by red arrows shows a bifurcation (panels 1, 2) and contact zone (panels 3, 4) on the one side, while the vessel indicated by blue arrows shows bifurcations (panels 1, 2, 6, 7) and contact zones (panels 3, 5) on both sides along the longitudinal axis. A possible topology of the latter vessel is shown in panel 8 (not in scale). (B) The vasculature marked with blue arrows shows a contact zone only in the central region (panels 2, 3) and not on both sides (panels 1, 4-6) along the longitudinal axis. A possible topology of the vessel is shown in panel 7 (not in scale). Scale bar: 50 μm. [file 1471-213X-10-84-S5.JPEG]

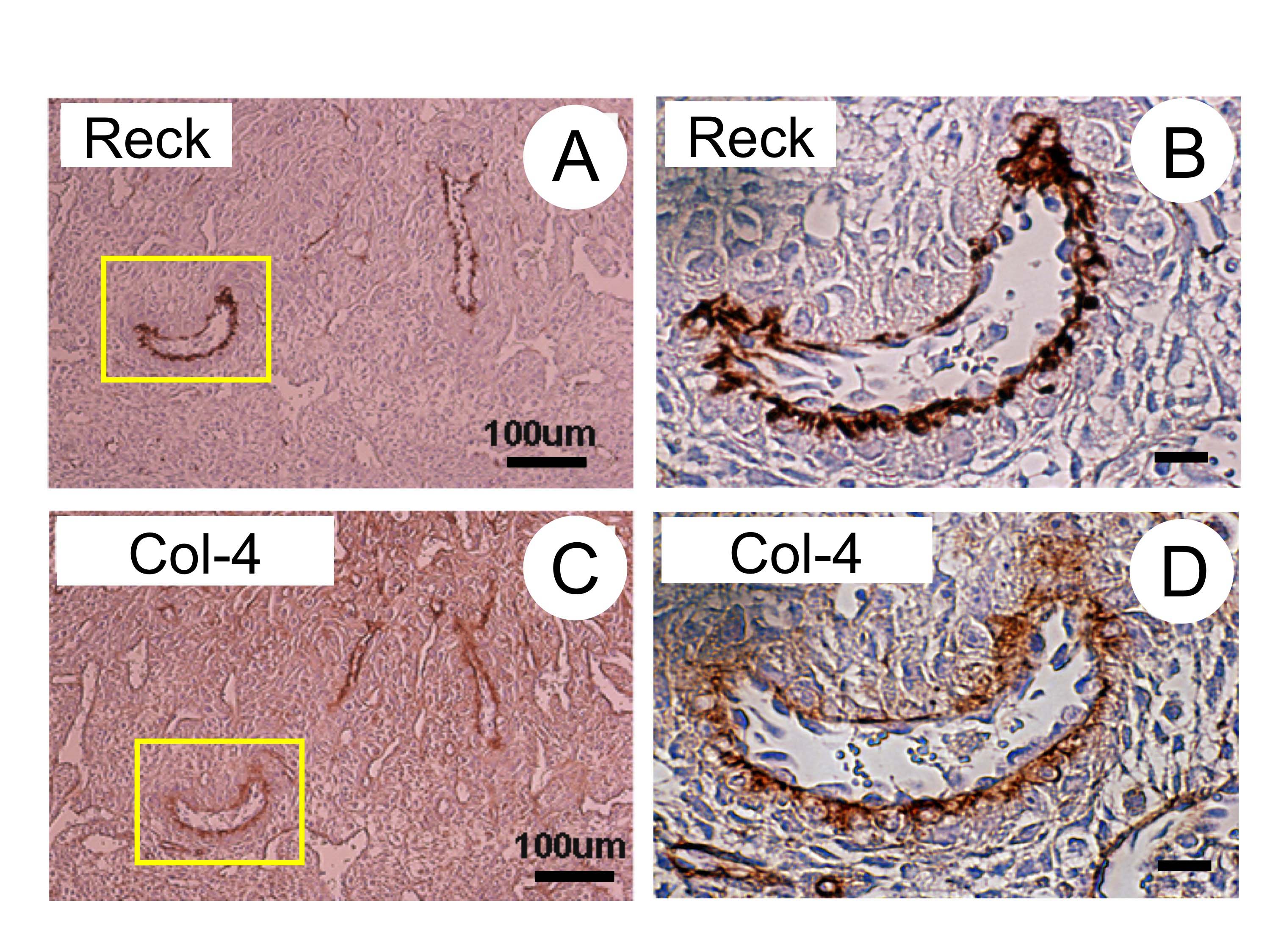

Supplement: Additional file 6 — Reck- and type IV collagen-immunoreactivity around blood vessels in the DB. DB vessels in two adjacent sections were stained for Reck (A, B) and type IV collagen (C, D). Magnified views of the area indicated by yellow boxes in (A) and (C) are shown in (B) and (D), respectively. Type IV collagen is abundant around the Reck-positive cells. Scale bar: A, C 100 μm; B, D 20 μm. [file 1471-213X-10-84-S6.JPEG]

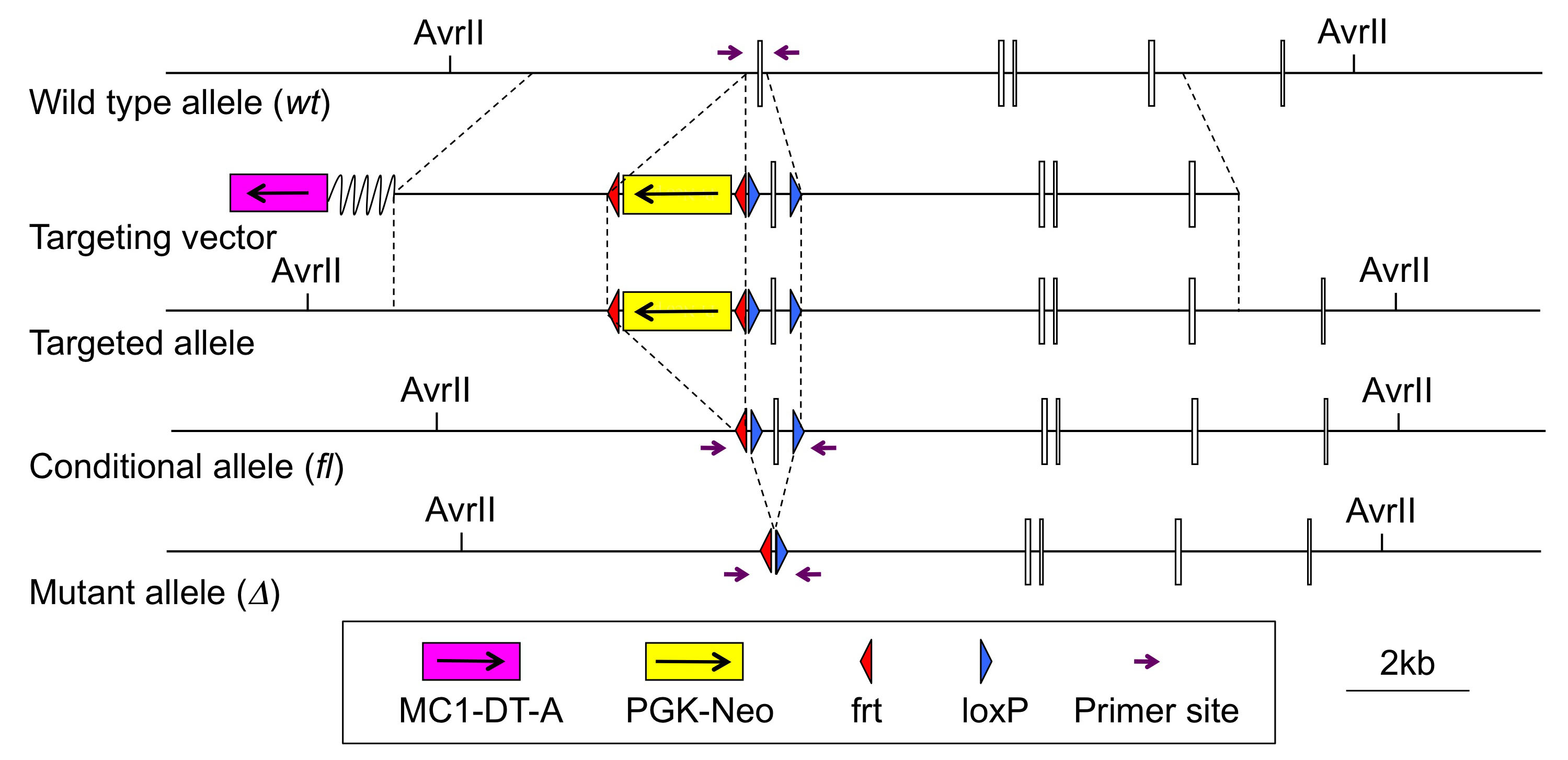

Supplement: Additional file 7 — Reck targeting strategy. See Methods for detail. PCR with primers AGTACATGACTTAGGAACAG (→) and AACTGCAATATCTGGGATAC (←) generates 853, 1252, and 721-bp products representing wt, fl, and Δ allele, respectively. [file 1471-213X-10-84-S7.JPEG]
